# Bibliometric Analysis of Global Research Trends on Ultrasound Microbubble: A Quickly Developing Field

**DOI:** 10.3389/fphar.2021.646626

**Published:** 2021-04-22

**Authors:** Haiyang Wu, Linjian Tong, Yulin Wang, Hua Yan, Zhiming Sun

**Affiliations:** ^1^ Clinical College of Neurology, Neurosurgery and Neurorehabilitation, Tianjin Medical University, Tianjin, China; ^2^ Tianjin Key Laboratory of Cerebral Vascular and Neurodegenerative Diseases, Tianjin Neurosurgical Institute, Tianjin Huanhu Hospital, Tianjin, China; ^3^ Department of Orthopaedic Surgery, Tianjin Huanhu Hospital, Tianjin, China

**Keywords:** ultrasound, microbubble, bibliometric analysis, research trends, hot spots

## Abstract

**Background:** Microbubbles are widely used as highly effective contrast agents to improve the diagnostic capability of ultrasound imaging. Mounting evidence suggests that ultrasound coupled with microbubbles has promising therapeutic applications in cancer, cardiovascular, and neurological disorders by acting as gene or drug carriers. The aim of this study was to identify the scientific output and activity related to ultrasound microbubble through bibliometric approaches.

**Methods:** The literature related to ultrasound microbubble published between 1998 and 2019 was identified and selected from the Science Citation Index Expanded of Web of Science Core Collection on February 21, 2021. The Scopus database was also searched to validate the results and provided as supplementary material. Quantitative variables including number of publications and citations, H-index, and journal citation reports were analyzed by using Microsoft Excel 2019 and GraphPad Prism 8.0 software. VOS viewer and CiteSpace V were used to perform coauthorship, citation, co-citation, and co-occurrence analysis for countries/regions, institutions, authors, and keywords.

**Results:** A total of 6088 publications from the WoSCC were included. The United States has made the largest contribution in this field, with the majority of publications (2090, 34.3%), citations (90,741, 46.6%), the highest H-index (138), and close collaborations with China and Canada. The most contributive institution was the University of Toronto. Professors De Jong N and Dayton P A have made great achievements in this field. However, the research cooperation between institutions and authors was relatively weak. All the studies could be divided into four clusters: “ultrasound diagnosis study,” “microbubbles’ characteristics study,” “gene therapy study,” and “drug delivery study.” The average appearing years (AAY) of keywords in the cluster “drug delivery study” was more recent than other clusters. For promising hot spots, “doxorubicin” showed a relatively latest AAY of 2015.49, followed by “nanoparticles” and “breast cancer.”

**Conclusion:** There has been an increasing amount of scientific output on ultrasound microbubble according to the global trends, and the United States is staying ahead in this field. Collaboration between research teams still needs to be strengthened. The focus gradually shifts from “ultrasound diagnosis study” to “drug delivery study.” It is recommended to pay attention to the latest hot spots, such as “doxorubicin,” “nanoparticles,” and “breast cancer.”

## Introduction

Ultrasound imaging is extensively used for diagnosis and efficacy evaluation in almost every medical discipline worldwide. The popularity of ultrasound imaging mainly derives from its relatively low cost, portability, and the lack of exposure to ionizing radiation (Akbari and Fei, 2012; Mirniaharikandehei et al., 2019). Typically, the ultrasound probe emits plane or diverging ultrasound waves, which can get reflected or scattered when passing through nonhomogeneous tissues and then detected by the same probe (Kiessling et al., 2009). Therefore, ultrasound imaging relies on the different scattering properties of healthy and unhealthy tissues. Since the malignant or benign lesions do not always provide adequate contrasts, intravenous injection of contrast agents may enhance our ability to discern the structures of interest (Frinking et al., 2000; Fan C. H. et al., 2014; Hunt and Romero, 2017).

The technique of contrast-enhanced ultrasound was first reported by Gramiak and Shah in 1968 (Gramiak and Shah, 1968; Gramiak et al., 1969). These early echocardiographers found that tiny air bubbles were occasionally introduced into the circulation when intravenous injection of agitated saline was carried out, resulting in transient echo enhancement of the aorta and ventricle (Becher et al., 1988; Blomley and Cosgrove, 1997). However, due to the instability and high solubility of these air bubbles in the blood, such in situ-generated microbubbles dissolve in a matter of seconds. More than 50 years after these first reports, the development of these techniques has been expanding, as reflected by the numerous clinical applications of ultrasound microbubble contrast agents available today (Lim et al., 2004; Mulvana et al., 2017; Cleve et al., 2019).

Ultrasonic contrast agents are characterized by a microbubble structure consisting of an inert gas core, surrounded by a biocompatible shell to act as blood pool agents (Becher et al., 1988; Lim et al., 2004; Mulvana et al., 2017; Espitalier et al., 2020). Various compositions have been applied to produce the encapsulating shell, including lipids, proteins, nonionic surfactants, biodegradable polymers, etc. (Sierra et al., 2017; Upadhyay and Dalvi, 2019). The major commercial inert gas core formulations have included air (Upadhyay and Dalvi, 2019), perfluorocarbons (Sharma et al., 2019), and sulfur hexafluoride (Tang et al., 2017). To date, there are a variety of commercial or noncommercial microbubble contrast agents, which all differ in their gas cores and encapsulating shells. With the advent of this technique, the diagnostic sensitivity and specificity of ultrasonic technology has further improved. The basic research and clinical application of microbubble contrast agents in a number of clinical specialties such as cardiac (Kurt et al., 2009), hepatic (Sidhu et al., 2004), renal (Grabner et al., 2016), and breast (Marshalek et al., 2016) imaging are also expanding.

Notably, in addition to ultrasonic diagnosis, with the development of ultrasound-targeted microbubble destruction (UTMD) technology (Wu et al., 2020) and the ultrasound microbubble carrying gene (UMCG) (Touahri et al., 2020), there are numerous other therapeutic applications of microbubbles, for example, as gene/drug carriers or delivery enhancers (Di et al., 2019; Touahri et al., 2020; Wu et al., 2020). The synergistic combination of microbubbles and ultrasound can result in a variety of dynamic reactions mainly determined by the amplitude of the ultrasound waves. At a low acoustic pressure level, microbubbles undergo oscillation, expansion, and contraction in response to the cyclic alternating pattern of the incident wave, which is also known as the stable cavitation effect. Stable oscillating bubbles scatter ultrasonic energy effectively, and the detection of scattered waves visualizes the distribution of bubbles in the vascular. Thus, a low acoustic pressure pattern is more suitable for diagnostic purposes rather than treatment.

However, the stable cavitation of microbubbles can transform to inertial cavitation at higher acoustic pressures. In this situation, inertial cavitation mainly presents as destruction and fragmentation of microbubbles accompanied with jetting, microstreaming, and a shock wave (Mehier-Humbert et al., 2005; Fan Z et al., 2014). These effects can increase the cell membrane permeability and promote cell endocytosis, thus providing a channel for the transmembrane delivery of drugs, genes, and other macromolecules by temporarily opening the tight junction of cells, the blood–brain barrier, or the blood–tumor barrier (Fan C. H. et al., 2014; Park et al., 2017). As a result, with the advantages of noninvasive, improved therapeutic targeting; ability to deliver many types of therapeutic cargo; and controlled drugs/genes release, ultrasound coupled with microbubbles has seen promising applications with a combined diagnostic and therapeutic role in cancer, cardiovascular diseases, and neurological disorders (Sidhu et al., 2004; Kurt et al., 2009; Grabner et al., 2016; Marshalek et al., 2016; Lipsman et al., 2018).

In recent years, the interest in ultrasound microbubble has drastically increased and many articles about ultrasound microbubbles have been published (Fan Z et al., 2014; Park et al., 2017; Lipsman et al., 2018). The rapid growth of ultrasound microbubble articles makes it difficult to identify the new developments and emerging trends in this area. To the best of our knowledge, the global research trend in ultrasound microbubbles has not been well studied yet.

Bibliometrics is the use of mathematical–statistical methods for summarizing scientific activities in a research field and further identifying research frontiers, hot spots, or rising patterns based on the literature databases and metrology characteristics (Chen et al., 2014; Hong et al., 2016). Meanwhile, besides characterizing and predicting development trends in a certain field, such an analysis can also be used to compare contributions across disparate countries, institutions, authors, and journals (Merigó et al., 2019; Laengle et al., 2020). Several visualization tools like CiteSpace (Synnestvedt et al., 2005), VOS viewer (van Eck and Waltman, 2010), and HistCite (Gu et al., 2019) have been developed to help researchers create knowledge maps, evaluate the latest cutting-edge research progress, and visualize the trends in scientific publications (Laengle et al., 2020; Modak et al., 2020). Until now, these tools have been used for estimating the research trends in various medical fields, such as potassium channels (Shi et al., 2020), exosomes (Wang et al., 2019), the mTOR signaling pathway (Fang et al., 2020), and nonmedical fields (Laengle et al., 2020; Modak et al., 2020).

Therefore, we collected both qualitative and quantitative data of publications and also identified the most productive/influential countries, institutions, authors, and journals based on the number of publications or citations in the field of ultrasound microbubble research. The objective of this study was to provide the first comprehensive bibliometric analysis of ultrasound microbubble for the 22-year period between 1998 and 2019 and also provide new insight for scholars who have entered or are about to enter this field on the current status, the emerging trends, and future research hot spots of ultrasound microbubble research from a global perspective.

## Methods

### Data Source

The Science Citation Index Expanded (SCI-Expanded) of Thomson Reuters’ Web of Science Core Collection (WoSCC) (searched at http://lib.tmu.edu.cn/) was used as the main data source. The WoSCC covers a considerable amount of high-quality scientific literature in the biomedical, natural, and social sciences, and it was used as the main data source. It is regarded as one of the most widely accepted and suitable databases for bibliometric analysis of scientific publications (Shi et al., 2020; Wang et al., 2019; Merigó et al., 2019; Chen et al., 2021). In addition, the Scopus database (searched at https://www.scopus.com/) was also searched to validate the results obtained from the WoSCC and provided as supplementary material. Scopus, developed by Elsevier, is a large abstract and citation database of scientific peer-reviewed literature, which contains more than 22,000 titles from international publishers and is also available for bibliometric research (Yeung et al., 2018; Romero and Portillo-Salido, 2019).

### Data Collection

**FIGURE 1:**
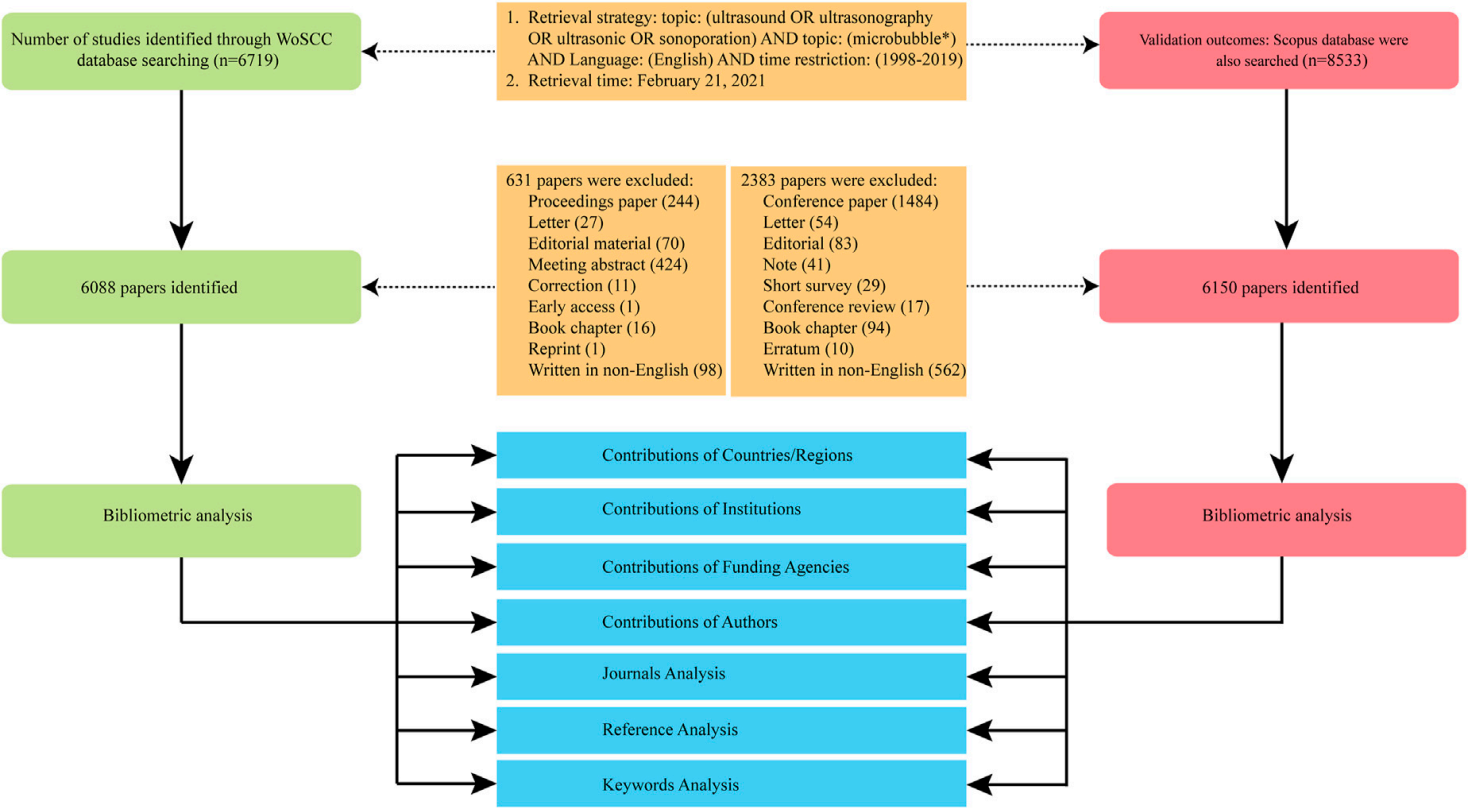
Flow diagram of literature filtering.

The literature regarding ultrasound microbubble was retrieved on a separate day (February 21, 2021), to avoid database update bias. The retrieval strategy was presented as follows: topic (ultrasound OR ultrasonography OR ultrasonic OR sonoporation) AND topic (microbubble*) AND Language (English). The document types were limited to original articles and reviews, and the time frame was from 1998 to 2019. All the retrieved papers from the WoSCC including the titles, keywords, author information, abstracts, and references were downloaded and saved in plain text format. The relevant information from the Scopus database was exported in CSV format (Figure 1).

### Data Extraction

These files were imported into Microsoft Excel 2019 (Microsoft Corporation, Redmond, Washington, WA, United States) for further data processing. Two researchers independently performed the literature selection, data extraction, and analysis to ensure the reliability of the results. Data extracted from the selected articles include the general information about annual number of publications, citation frequency, original countries, authors, journals, institutions, and funding agencies. The journal impact factor (IF) and category (Q1, Q2, Q3, and Q4) of each journal were retrieved from the Journal Citation Reports (JCR) 2019 (available at: http://thomsonreuters.com/journal-citation-reports/), which is the most frequently used reference standard for evaluating the journal performance within its field. H-index was another important indicator to evaluate the scientific output and academic status of a researcher. It is also considered a useful indicator for evaluating the productivity and impact of a country, institution, or journal (Engqvist and Frommen, 2008). In this process, any discrepancies between the two researchers were discussed to reach consensus. Then, data cleaning and analysis was conducted manually in Excel.

### Data Visualization and Analysis

Microsoft Excel 2019 and GraphPad Prism 8.0 (GraphPad Software Inc.) software were used for descriptive statistical analysis of publications including number of publications, citation frequency, original countries, authors, journals, institutions, H-index, and funding agencies.

The Java program VOS viewer (van-Eck and Waltman, 2010, Netherlands, downloaded from http://vosviewer.com) is a software tool used for constructing visualization networks of scientific literature (van-Eck and Waltman, 2010). These networks include researchers, journals, research organizations or countries and can be connected by coauthorship, citation, co-citation, and co-occurrence analysis. Coauthorship analysis is a mature measure to establish similarity relationships among items through the number of coauthored documents. Citation analysis of items is created based on the number of times they cited each other. Co-citation and co-occurrence analysis illustrate the relationship among items, respectively, based on the number of times they are referenced together and the number of works where they occur together (Leydesdorff et al., 2013). In this study, this software was used for i) country/region citation analysis; ii) institution citation analysis; iii) author coauthorship and co-citation analysis; iv) journal co-citation analysis; and v) keyword co-occurrence analysis.

In the network graphs generated by the VOS viewer, each node represents a different parameter, such as countries/regions, institutions, journals, or keywords. Nodes were given different colors according to different taxonomies or occurrence times. In general, node size was determined by the calculated betweenness centrality of each parameter, with bigger nodes representing a higher level of centrality. The links between the nodes indicated correlation between parameters, and the thickness of the links represented the strength of links. Total link strength (TLS) was used to quantitatively assess the links.

In addition to VOS viewer software, we also used another piece of Java-based scientometrics research software, CiteSpace V (version 5.7 R2, Chen et al., 2014, Drexel University, United States, downloaded from http://cluster.ischool.drexel.edu/∼cchen/citespace/download), to perform i) institution coauthorship analysis; ii) author coauthorship and co-citation analysis; iii) journal co-citation analysis; iv) a dual-map overlay of journals; and v) reference co-citation analysis (Synnestvedt et al., 2005).

In the created visualization maps, each node represents the type of study being analyzed, and its size is proportional to the number of occurrences or citations. Links between nodes represent the strength of collaborations, co-citations, or co-occurrence. The color of each node represents the distribution time. Clusters formed by neighbor nodes correspond to related topics, and the flow of knowledge between clusters can be seen in the change of color.

Different parameter settings in the VOS viewer or CiteSpace will change the analysis result of visualization. Some of the key parameter settings of VOS viewer software have been described in the “Results” section. The parameter settings of CiteSpace software were as follows: time slicing (from 1998 to 2019), years per slice (one- or two-year), term source (title, abstract, author keyword, and keyword plus), node type (choose one parameter at a time such as institution, author, cited author, reference, or keyword), selection criteria (top 50), pruning (minimum spanning tree and pruning sliced networks), and visualization (cluster view or time zone view). A more detailed description of the software can be found in the operational manual (http://cluster.ischool.drexel.edu/∼cchen/citespace/CiteSpaceManual). In addition, a free online analysis platform of literature metrology (http://bibliometric.com) was also available for data visualization.

Data from the WoSCC was analyzed using the VOS viewer, Citespace, and the online analysis platform. All these results are presented in the main text. We also used the software of the VOS viewer to visualize the data derived from Scopus, and the detailed results are described in the Supplementary Material.

### Research Ethics

No ethical approval is required since the data used in this manuscript were downloaded from public databases and did not involve animal/human samples.

## Results

### Trend of Global Publications and Citations

**FIGURE 2:**
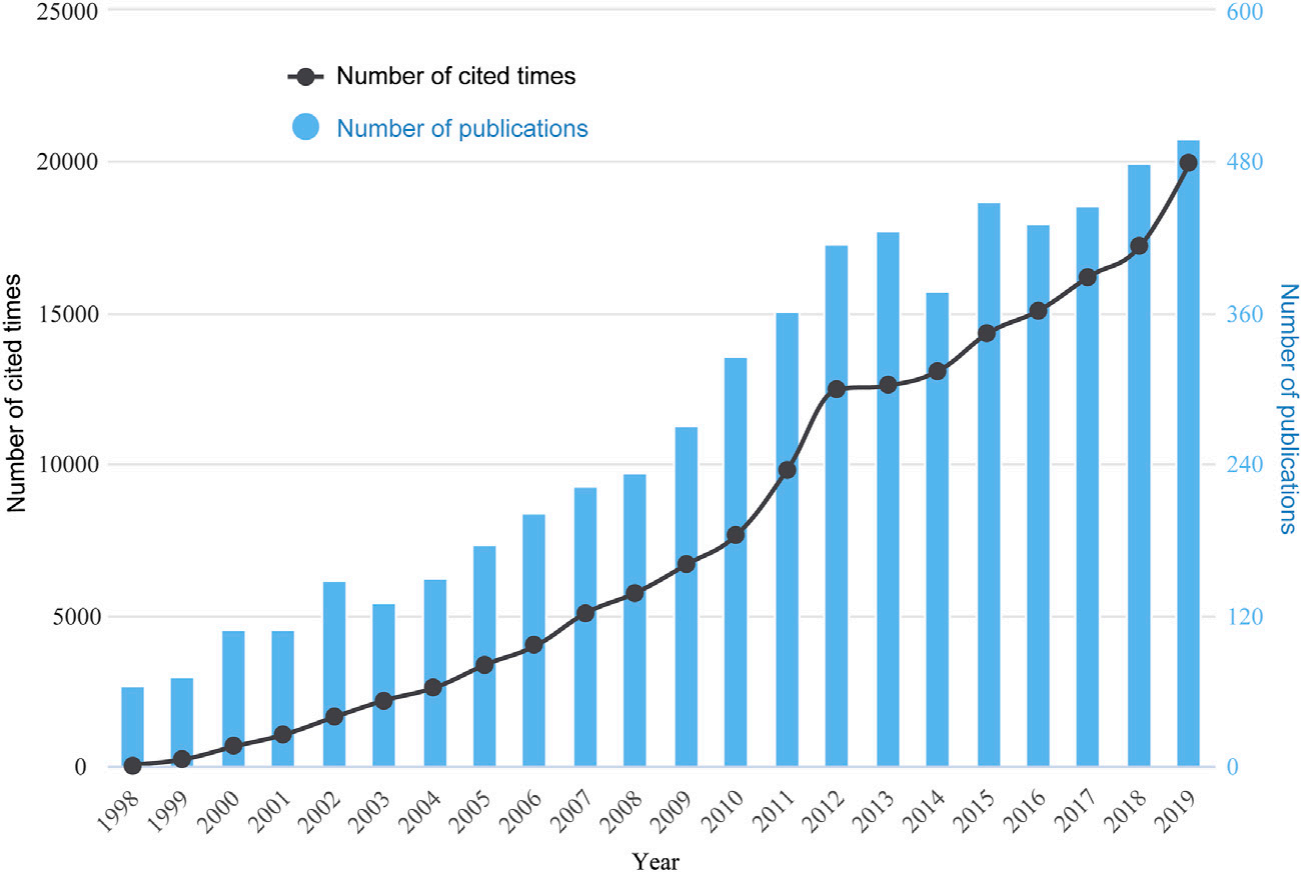
Global trend of annual publications and citations related to ultrasound microbubble research from 1998 to 2019.

A total of 6088 publications obtained from the WoSCC including 5572 articles and 516 reviews met the inclusion criteria from 1988 to 2019 (Figure 1). As shown in Figure 2 and Supplementary Figure S1A, the trend of global ultrasound microbubble research publications has been steadily increasing in the past 22 years. The number has increased from 64 (1998) to 498 (2019), and almost 38% of them (2301) were published in the last five years. All the publications have been cited 194,896 times, and each paper was cited 32 times on average.

### Contributions of Countries/Regions

**FIGURE 3:**
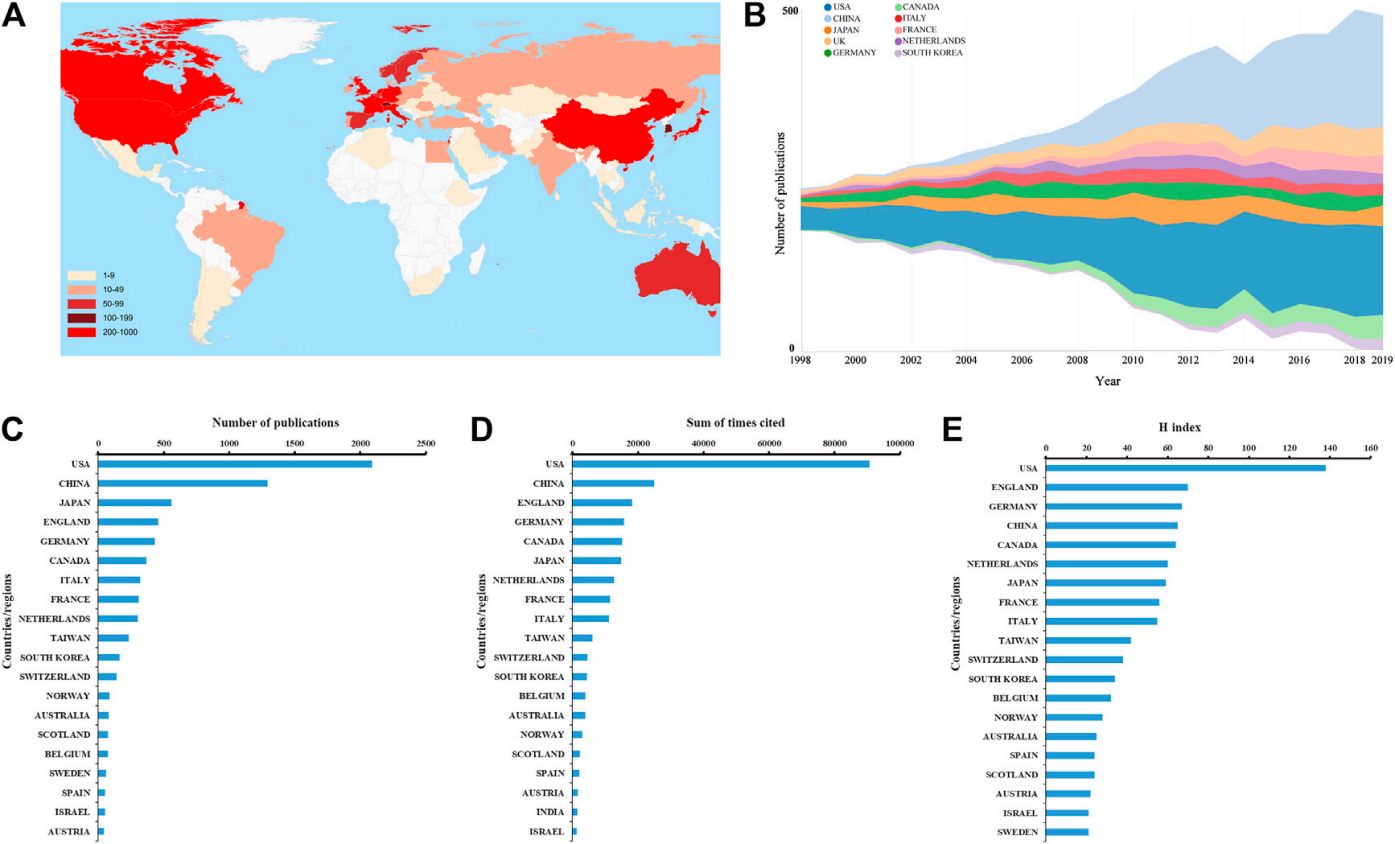
**(A)** World map displaying the global distribution of ultrasound microbubble research. Different countries were denoted with different colors based on the number of articles published. **(B)** Growth trends in the publication quantity of the top 10 countries/regions in ultrasound microbubble research from 1998 to 2019. **(C–E)** Total number of publications, sum of total citations, and H-index of top 20 countries in this field.

The distribution of published articles was shown on a world map. Colors on the map represent different density values (Figure 3A). Figure 3B displays a transformative trend in the annual publication numbers of the top 10 countries from 1998 to 2019. A total of 73 countries/regions have participated in the publication. The United States was the foremost productive country, with 2,090 papers published (34.3%), followed by China (1,293, 21.2%) (Figure 3C and Supplementary Figure S1B). As presented in Figures 3D,E, the total citations (90,741 times) and H-index (138) in the United States exceeded other countries/regions, ranking first in the world.

**FIGURE 4:**
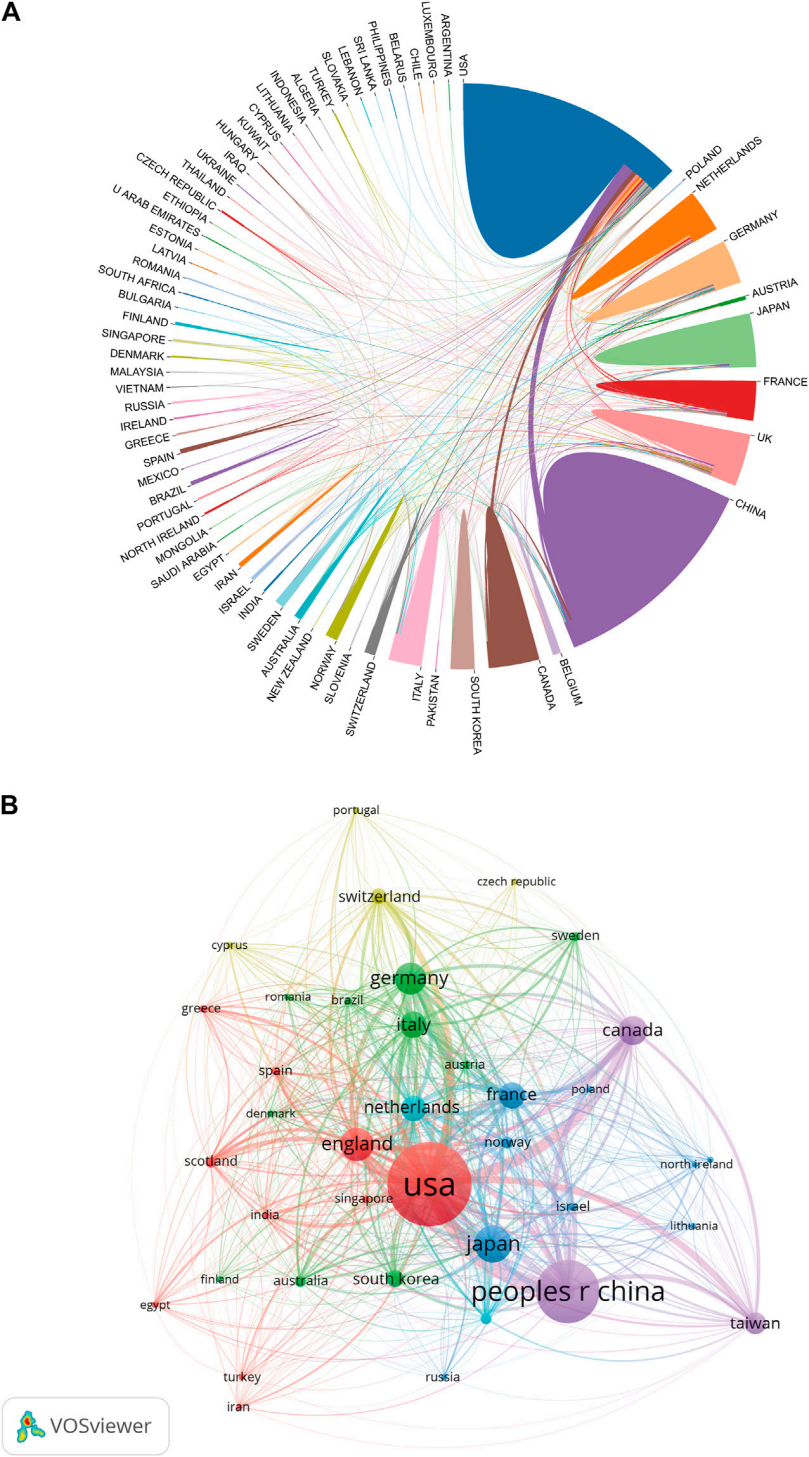
**(A)** Distribution and international cooperation of countries/regions that are involved in ultrasound microbubble research. The thickness of the line reflects the frequency of the cooperation. The thicker the line, the stronger the cooperation. **(B)** Citation map of countries/regions on ultrasound microbubble research generated by the VOS viewer. Each node represents a country/region, and node size indicates the number of publications. The connection between the nodes represents a citation relationship, and the thickness of the lines indicates citation strength (weights on the TLS).

As for country/region coauthorship analysis, the United States was at the center of research on ultrasound microbubble and had close collaborations with China and Canada (Figure 4A). Publications originating from 38 countries were selected, with the minimum number of documents from each country more than 10 and analyzed by using the VOS viewer (Figure 4B). There were 38 nodes and 620 links in the network map. The top three countries with the largest TLS were the United States (TLS = 43,891), China (TLS = 20,242), and England (TLS = 13,701).

### Contributions of Institutions

A total of 3056 institutions contributed to the publications on ultrasound microbubble research. The University of Toronto was the largest contributor in terms of numbers of publications with 240 papers, followed by the University of Virginia and Chongqing Medical University, with 176 and 159 papers, respectively. The top 10 most influential institutions and the quantity of articles in each institution are presented in Table 1. The top 20 institutions contributed to the total publications based on data obtained from the Scopus database was provided in Supplementary Figure S1C.

**TABLE 1:** Top 10 institutes that contributed to publications about ultrasound microbubble.

Rank	Institutions	Countries/regions	Count
1	University of Toronto	Canada	240
2	University of Virginia	United States	176
3	Chongqing Medical University	Mainland China	159
4	Chinese Academy of Sciences	Mainland China	126
5	Shanghai Jiao Tong University	Mainland China	125
6	University of Twente	Netherlands	124
7	Erasmus MC	Netherlands	121
8	University of Michigan	United States	107
9	University of California, San Diego	United States	102
10	Univ london imperial coll sci technol med	England	101

**FIGURE 5:**
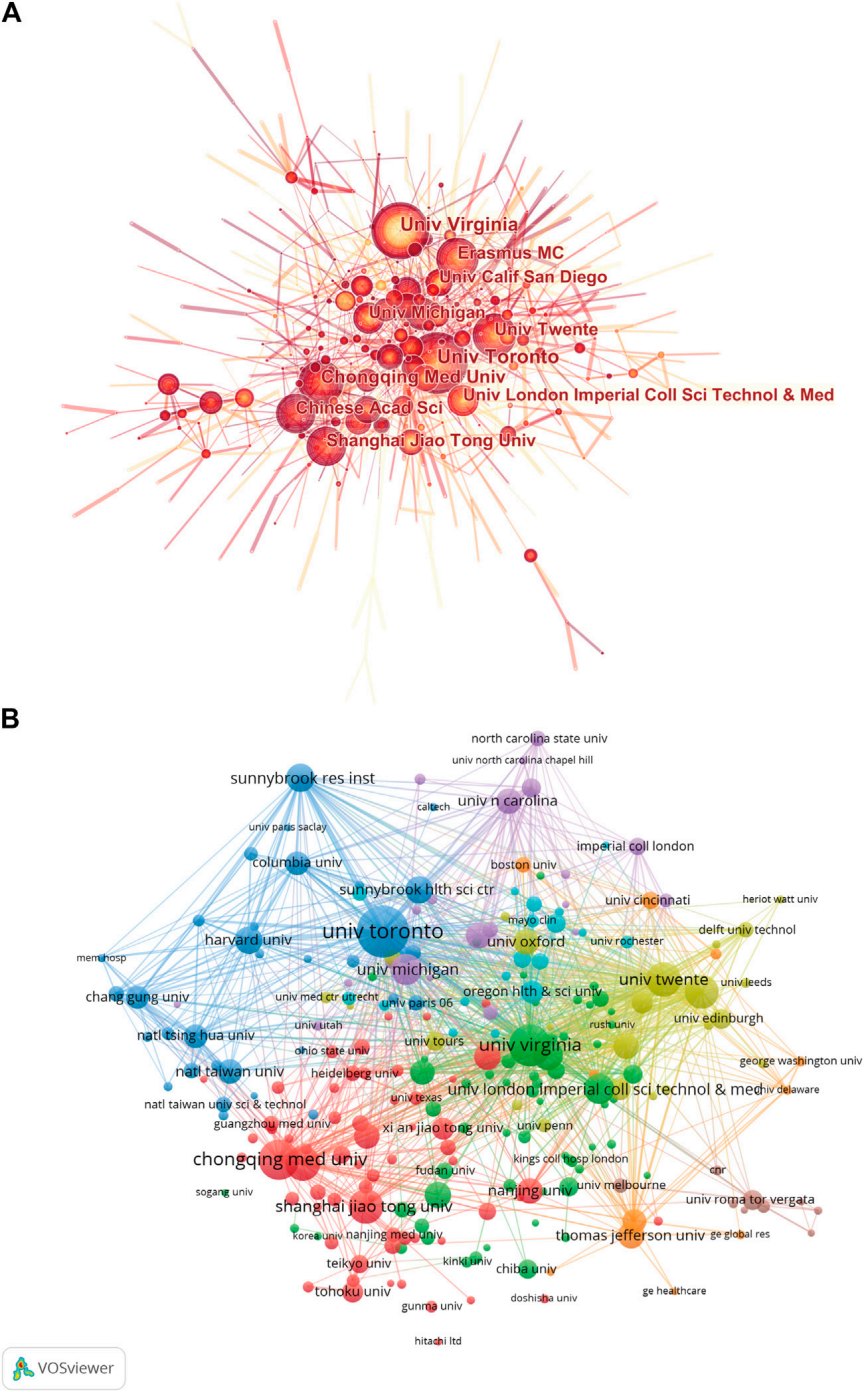
**(A)** Network map of institution coauthorship analysis based on CiteSpace. In the visualization map, each node represents an institution, and its size is proportional to the number of publications. Links between nodes represent the strength of collaborations. **(B)** Mapping of the citation analysis among 238 identified institutions on ultrasound microbubble research based on the VOS viewer. The explanation of the nodes and links in the map is identical to that described in Figure 4B above.

As shown in Figure 5A, the network map of cooperation relationships between institutions was a low-density map (density = 0.0056). The centrality indexes in most institutions were less than 0.15. As for the institution citation analysis shown in Figure 5B, only institutions with a minimum of 10 publications were included. There were 238 nodes and 16,471 links in the network map. The top three institutions with the largest TLS were the University of Virginia (TLS = 11,891), the University of Toronto (TLS = 11,379), and the University of Twente (TLS = 9,492).

### Contributions of Funding Agencies

Table 2 lists the world’s top 10 funding agencies which sponsored the output of ultrasound microbubble research. Among them, 5 agencies were from the United States. The United States Department of Health and Human Services (HHS) ranked first, supporting the highest number of 1,257 studies. The National Institutes of Health (NIH) ranked second (1,256), and the National Natural Science Foundation of China (NSFC) ranked third (763). Supplementary Figure S1D also provides the top 20 research funds and the number of publications in each fund based on the Scopus database.

**TABLE 2:** Top 10 related funding agencies.

Funding agencies	Countries/regions	Count	Percentage (N/6088)
United States Department of Health and Human Services (HHS)	United States	1257	20.65
National Institutes of Health (NIH)	United States	1256	20.63
National Natural Science Foundation of China (NSFC)	China	763	12.53
NIH National Institute of Biomedical Imaging and Bioengineering (NIBIB)	United States	510	8.38
NIH National Cancer Institute (NCI)	United States	440	7.28
NIH National Heart, Lung, and Blood Institute (NHLBI)	United States	351	5.78
Ministry of Education, Culture, Sports, Science, and Technology (MEXT)	Japan	243	3.99
Japan Society for the Promotion of Science	Japan	223	3.66
Grants in Aid for Scientific Research (KAKENHI)	Japan	196	3.22
United Kingdom Research Innovation (UKRI)	United Kingdom	186	3.06

### Contributions of Authors

**FIGURE 6:**
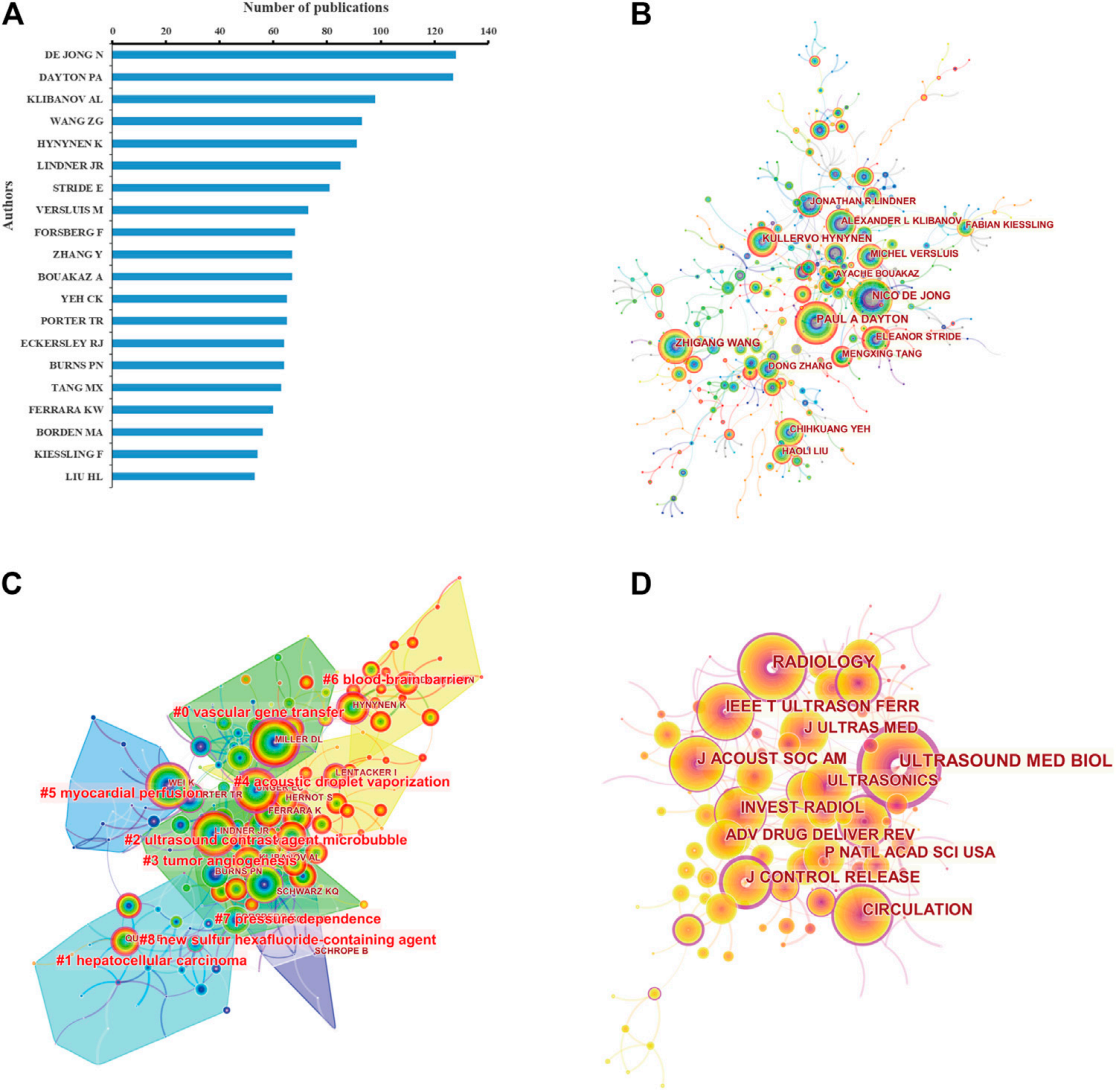
**(A)** Top 20 most productive authors based on the number of publications. Coauthorship **(B)** and cocitation **(C)** analysis of authors involved in ultrasound microbubble research based on CiteSpace. Cited authors with similar categories are gathered in a cluster. All the clusters are labeled in red text, and the links between nodes represent authors cited together. **(D)** Cocitation analysis of journals. The size of the nodes represents the number of total cited times of the journals. The larger the node is, the more the cited times of the journal is. Nodes with purple outer rings are journals with high centrality (more than 0.1) in the network.

Figure 6A and Supplementary Figure S2A list the top 20 authors who published the greatest number of papers. From the WoSCC, a total of 1,522 publications by the top 20 authors accounted for 25% of all literature in this field. De Jong N from the Netherlands was the author with the most publications of 128, followed by Dayton P A from the United States with 127 papers, Klibanov A L from the United States with 98 papers, and Wang Z G from China with 93 papers.

As shown in Figure 6B, the author coauthorship network map was a low-density map (density = 0.0022). Dayton P A, De Jong N, and Wang Z G were located at a central position of the cooperating clusters. The co-citation network between authors was also analyzed (Figure 6C). In the cluster map, there were 182 nodes and 9 co-cited author clusters. Lindner J R owned the highest centrality. The silhouette value of clusters #0 to #8 was from 0.759 to 0.985, showing good homogeneity. Authors’ research categories of the 9 clusters were “vascular gene transfer” (#0), “hepatocellular carcinoma” (#1), “ultrasound contrast agent microbubble” (#2), “tumor angiogenesis” (#3), “acoustic droplet vaporization” (#4), “myocardial perfusion” (#5), “blood–brain barrier” (#6), “pressure dependence” (#7), and “new sulfur hexafluoride–containing agent” (#8). Similar results of author coauthorship and co-citation analysis were also obtained based on the Scopus database (Supplementary Figures S2B,C).

### Journal Analysis

In total, 1,053 journals have emerged recently in this research field. The top 10 active journals published 1,838 papers on ultrasound microbubble, accounting for 30.19% of all 6,088 publications (Table 3 and Supplementary Figure S3A). *Ultrasound in Medicine and Biology* published the most papers (690), accounting for 11.33% of the publications. *IEEE Transactions on Ultrasonics, Ferroelectrics, and Frequency Control* ranked the second, with 192 publications. *Theranostics* has the largest impact factor of 8.579, followed by *Radiology* (7.931) and the *Journal of Controlled Release* (7.727). According to the JCR 2019 standards, the top 10 most active journals were classified as Q1 in 6 and Q2 in 4.

**TABLE 3:** Top 10 journals in the field of ultrasound microbubble research ranked by publication number.

Rank	Journal title	Country	Count	Percentage (N/6088) (%)	IF (2019)	Quartile in category (2019)	H-index
1	*Ultrasound in Medicine and Biology*	United States	690	11.33	2.514	Q1	76
2	*IEEE Transactions on Ultrasonics, Ferroelectrics, and Frequency Control*	United States	192	3.15	2.812	Q1	39
3	*Journal of Controlled release*	Netherlands	154	2.53	7.727	Q1	57
4	*Journal of the Acoustical Society of America*	United States	149	2.45	1.78	Q2	39
5	*Journal of Ultrasound in Medicine*	United States	142	2.34	1.759	Q2	32
6	*Ultrasonics*	Netherlands	128	2.10	3.065	Q1	29
7	*Physics in Medicine and Biology*	England	103	1.69	2.883	Q2	31
8	*Plos One*	United States	98	1.61	2.74	Q2	25
9	*Radiology*	United States	98	1.61	7.931	Q1	52
10	*Theranostics*	Australia	84	1.38	8.579	Q1	23

The cocitation relationship among different journals was visualized in a cocitation network (Figure 6D and Supplementary Figure S3B). There were 135 nodes and 253 links in the co-cited network map in Figure 6D. *Ultrasound in Medicine and Biology* had the highest centrality, with a central value of 0.67, followed by the *Journal of Controlled Release* (0.56), *Radiology* (0.4), and *Circulation* (0.4).

**FIGURE 7:**
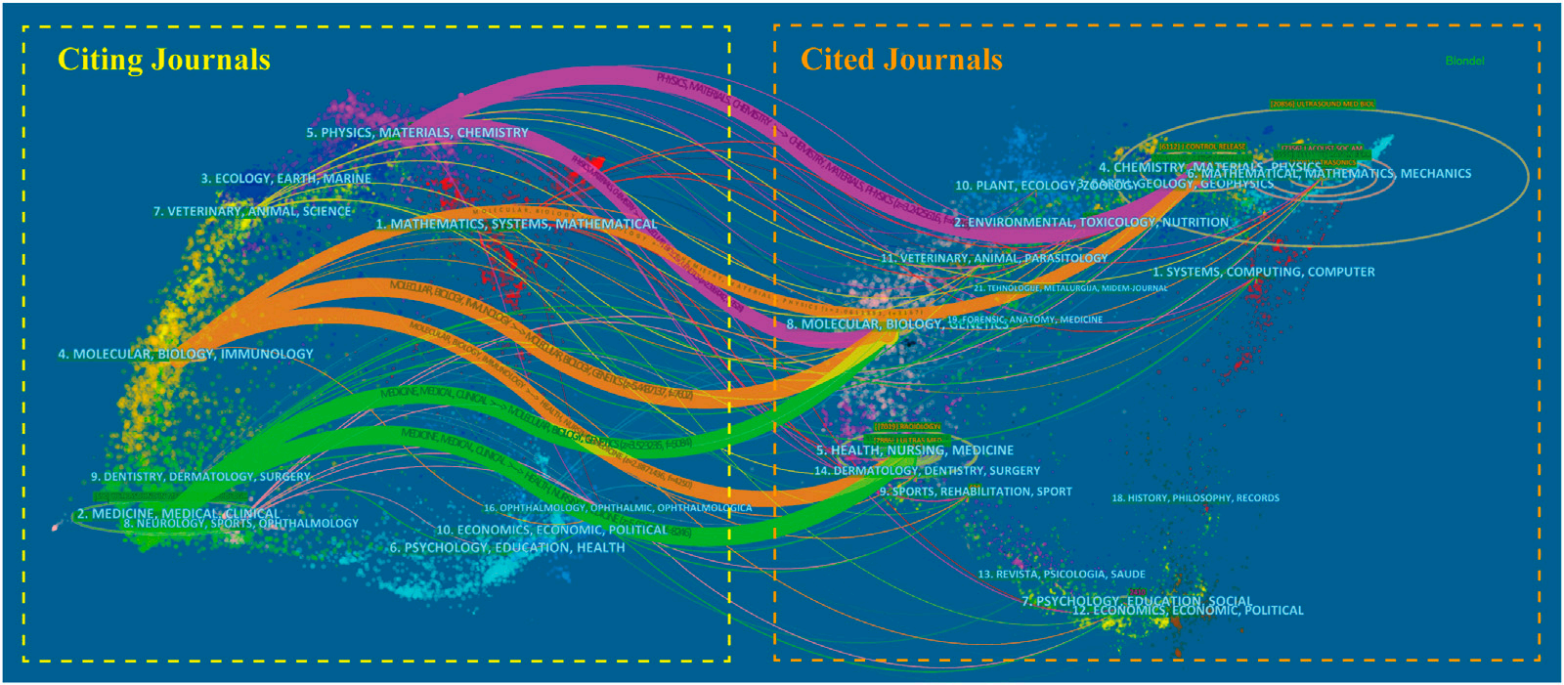
Dual-map overlay of the journals on ultrasound microbubble research generated by CiteSpace. The labels represent different research subjects covered by the journals. The citing journals are on the left side, while the other side of the map represents the cited journals. Different colored lines correspond to the different paths of references, beginning with the citing map and ending at the cited map. The path widths are scaled proportionally to the frequency of *z*-score-scale citation.

Figure 7 shows a dual-map overlay of the journals on ultrasound microbubble research. Collectively, there were seven main citation paths in the current map. The published studies mainly targeted journals in three fields: i) physics, materials, and chemistry; ii) molecular, biology, and immunology; and iii) medicine, medical, and clinical, whereas the most cited publications originated from the journals of i) chemistry, materials, and physics; ii) molecular, biology, and genetics; and iii) health, nursing, and medicine.

### Reference Analysis

Table 4 lists the basic information for the papers that were the top 10 most cited. These highly cited studies were published between 1998 and 2013, and 7 studies were published prior to 2010. Nine papers were coauthored, and the average number of authors per paper was 5.9. Additionally, four publications were completed through collaboration between institutes. The most highly cited paper was written by Wei et al. (1998) with 1,221 citations. The paper written by Ferrara et al. (2007) ranked second with 797 citations. The third position was occupied by Janib et al. (2010) with 772 citations.

**TABLE 4:** Top 10 ultrasound microbubble papers with the most citations.

Title	Journal	First author	Year	Citations
Quantification of myocardial blood flow with ultrasound-induced destruction of microbubbles administered as a constant venous infusion	*Circulation*	Wei K	1998	1221
Ultrasound microbubble contrast agents: Fundamentals and application to gene and drug delivery	*Annual Review of Biomedical Engineering*	Ferrara, Katherine	2007	797
Imaging and drug delivery using theranostic nanoparticles	*Advanced Drug Delivery Reviews*	Janib siti M	2010	772
Microbubbles in ultrasound-triggered drug and gene delivery	*Advanced Drug Delivery Reviews*	Hernot, Sophie	2008	612
Microbubbles in medical imaging: Current applications and future directions	*Nature Reviews Drug Discovery*	Lindner, JR	2004	569
Controlled vesicle deformation and lysis by single oscillating bubbles	*Nature*	Marmottant, P	2003	567
Complications of radiofrequency coagulation of liver tumors	*British Journal of Surgery*	Mulier, S	2002	521
Pulse inversion Doppler: A new method for detecting nonlinear echoes from microbubble contrast agents	*IEEE Transactions on Ultrasonics Ferroelectrics and Frequency*	Simpson, DH	1999	492
Guidelines and good clinical practice recommendations for contrast-enhanced ultrasound (CEUS) in the liver - update 2012 a WFUMB-EFSUMB initiative in cooperation with representatives of AFSUMB, AIUM, ASUM, FLAUS and ICUS	*Ultrasound in Medicine and Biology*	Claudon, Michel	2013	455
An acoustic rectifier	*Nature Materials*	Liang B	2010	431

**FIGURE 8:**
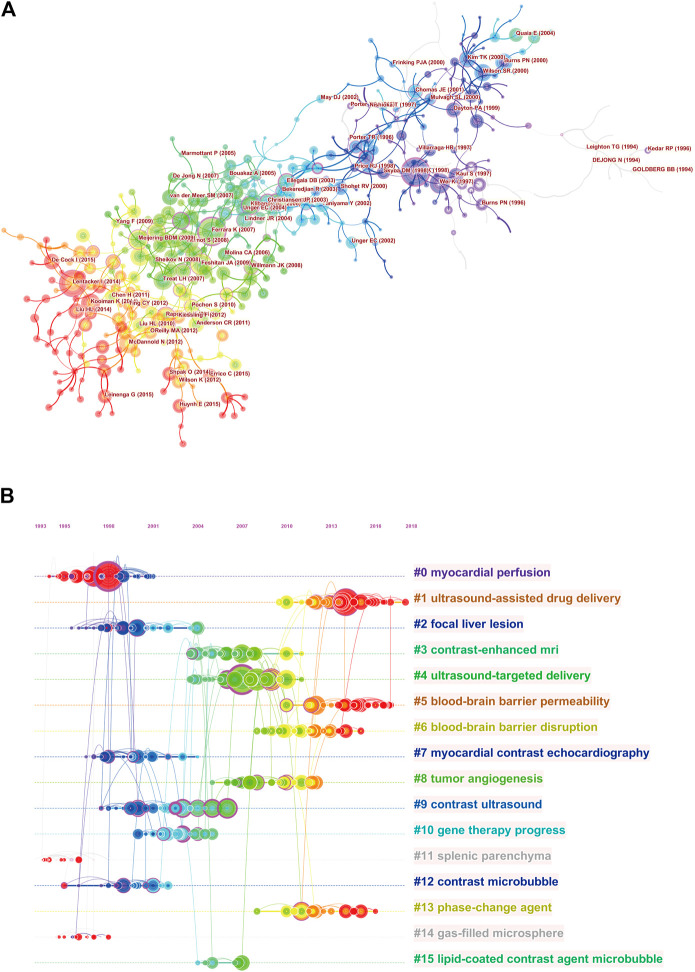
Cluster view **(A)** and timeline view **(B)** of cocitation reference. The clusters are arranged vertically in descending order according to their size. Sixteen major clusters are labeled and color-coded on the right. The time evolution is shown with different colored lines. The nodes on the lines indicate the references cited, and the links indicate the references cited together. The density of nodes at different time periods can reflect the dynamic changes of the corresponding clusters on the time axis.

The reference co-citation relationship was visualized in a co-citation network (Figure 8A and Supplementary Figure S4). As shown in Figure 8A, the co-citation network consists of 441 nodes and could be clustered into 16 main subclusters. The Q value of modularity is a measure to assess the significance of the community structure. The maximum Q value equal to or more than 0.3 indicates a significant community structure. In this study, modularity Q was 0.8144, indicating that the clusters of networks were reasonable. The silhouette value from clusters #0 to #14 was all more than 0.8, indicating the good homogeneity of the clusters. Figure 8B also shows the timeline view of the reference co-citation clusters, which could reflect the temporal characteristics of research hot spots in this field. The largest cluster was “myocardial perfusion” (#0), followed by “ultrasound-assisted drug delivery” (#1) and “focal liver lesion” (#2). The development of cluster 0 (myocardial perfusion) and cluster 11 (splenic parenchyma) occurred earliest, suggesting that early considerations focused on the diagnostic value of ultrasound microbubble. Cluster 1 (ultrasound-assisted drug delivery) and cluster 5 (blood–brain barrier permeability) are current research hot spots, which indicates that more concerns are shifting to potential therapeutic applications.

### Keyword Analysis

**FIGURE 9:**
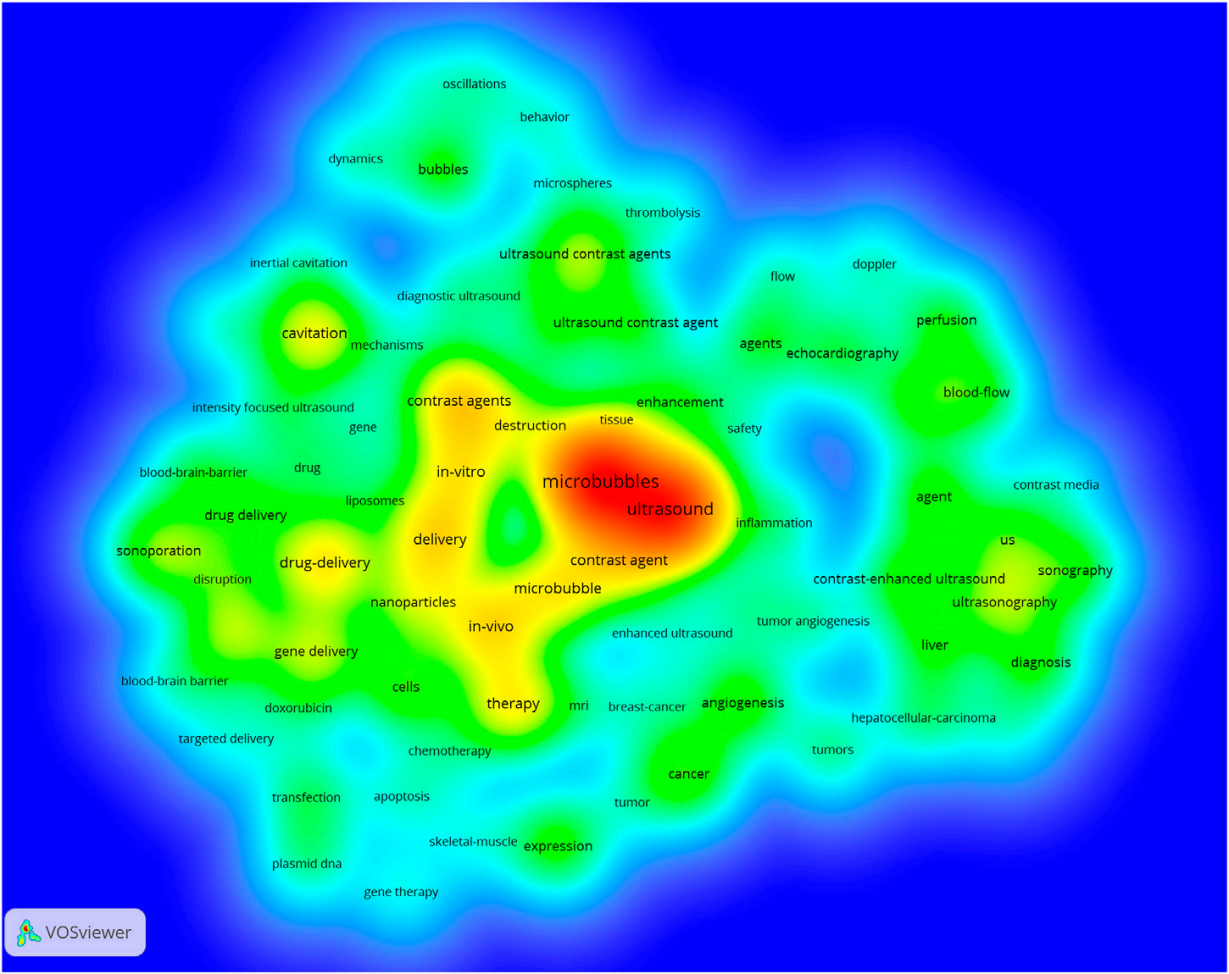
Density map of keywords generated by the VOS viewer. The deeper the color of a node, the more frequently keywords appear.

The goal of keywords co-occurrence analysis is to determine developing trends and hot topics, and it is one of the important approaches for tracing scientific development. A density map of high-frequency keywords was created using the VOS viewer. The results revealed that there were 14,325 keywords in the 6,088 papers, and 89 keywords appeared 100 times or more (Figure 9 and Supplementary Figure S5A). Among these, the top 20 keywords from the WoSCC in terms of occurrence frequency are listed in Table 5. Microbubbles and ultrasound were the most frequent keywords, with 2,804 and 2,071 co-occurrences, respectively, which were consistent with our research theme. Concerning the other keywords, some were related to ultrasound diagnosis such as contrast agents, ultrasound contrast agents, and focused ultrasound. Others were related to therapies such as drug delivery, therapy, gene delivery, and cancer.

**TABLE 5:** Top 20 keywords in terms of frequency.

Rank	Keyword	Occurrence	TLS	Rank	Keyword	Occurrence	TLS
1	Microbubbles	2804	2727	11	Sonoporation	414	410
2	Ultrasound	2071	1999	12	Ultrasound contrast agents	410	387
3	Contrast agents	1244	1219	13	Focused ultrasound	390	387
4	Delivery	656	644	14	Nanoparticles	368	365
5	*In vivo*	643	626	15	Cancer	355	352
6	Drug delivery	616	613	16	Ultrasonography	356	345
7	Therapy	563	560	17	Cells	338	337
8	Cavitation	534	517	18	Destruction	336	334
9	*In vitro*	511	504	19	Angiogenesis	324	319
10	Gene delivery	415	409	20	Expression	315	310

TLS: total link strength.

**FIGURE 10:**
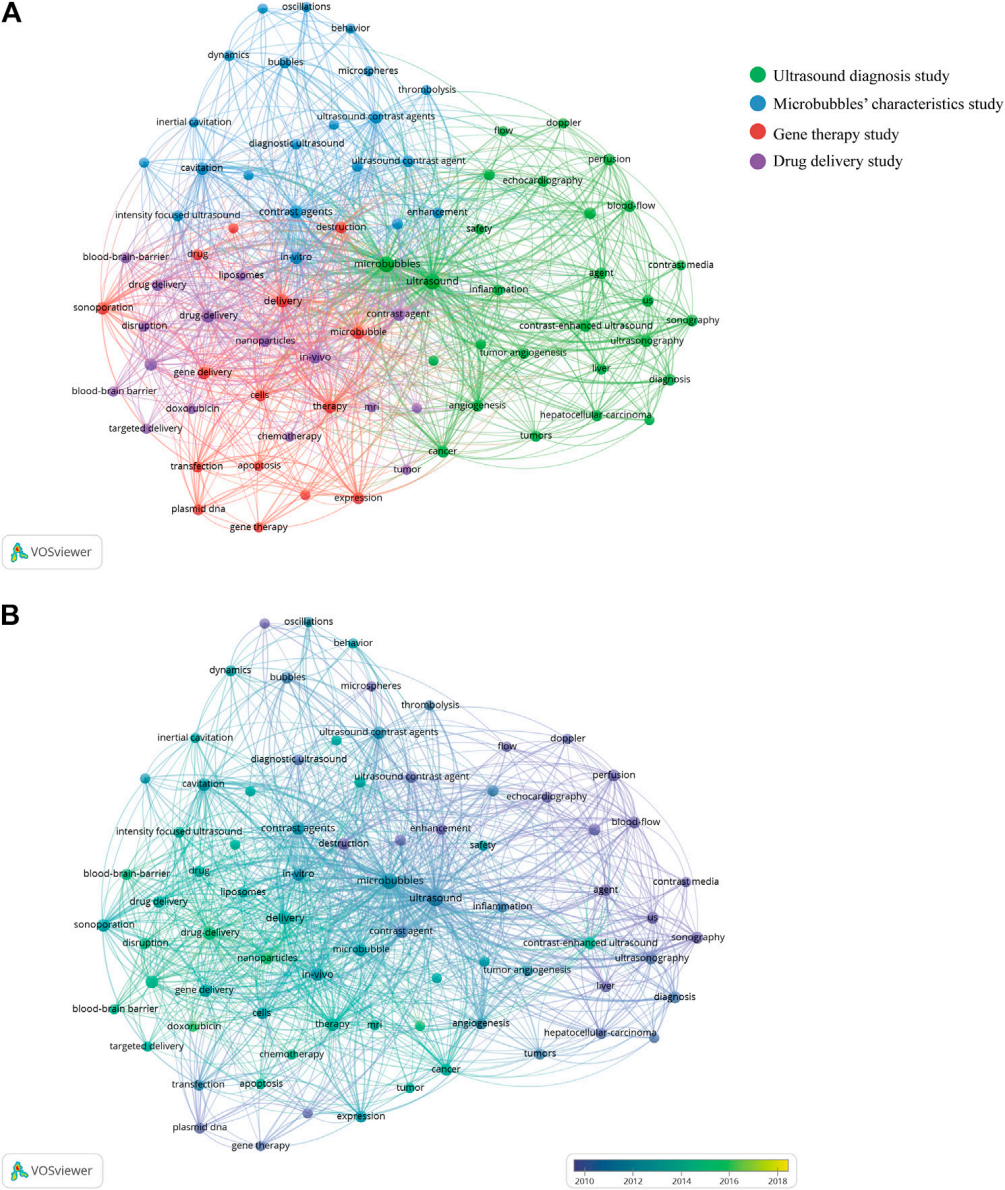
**(A)** Keyword cooccurrence analysis on ultrasound microbubble research using the VOS viewer. **(B)** Overlay visualization of the cooccurrence analysis. The purple nodes represent the keywords appearing earlier, whereas the yellow nodes reflect the recent occurrence.

As illustrated in Figure 10A, all the identified keywords could be divided into 4 clusters: “ultrasound diagnosis study,” “microbubbles’ characteristics study,” “gene therapy study,” and “drug delivery study”. These clusters showed the most prominent topics in ultrasound microbubble research so far. For the “ultrasound diagnosis study” cluster, the primary keywords were ultrasound, microbubbles, diagnosis, contrast-enhanced ultrasound, sonography, and doppler. As for the “microbubbles’ characteristics study” cluster, the frequently used keywords were contrast agents, cavitation, dynamics, ultrasound contrast agents, and behavior. In the cluster of “gene therapy study”, the prominent keywords were gene delivery, delivery, therapy, sonoporation, and expression. In cluster 4, the primary keywords were drug delivery, focused ultrasound, *in vivo*, nanoparticles, and chemotherapy. Similar results were also obtained by analyzing the Scopus database in Supplementary Figure S5B. It is noteworthy that the keywords related to gene therapy and drug delivery were combined into one cluster in this result, both of which were about therapeutic applications of ultrasound microbubble.

As shown in the overlay visualization map, different colors were applied for each keyword based on their average appearing year (AAY) (Figure 10B). Early research prior to 2010, this field is mainly focused on “ultrasound diagnosis study” and “microbubbles’ characteristics study”. Keywords in the “drug delivery study”–related cluster had the smallest AAY of all the clusters, which revealed that research studies in this direction have gained considerable attention and research focus recently. Meanwhile, the keywords “doxorubicin,” “nanoparticles,” and “breast cancer” showed a relatively latest AAY of 2015.49, 2015.35, and 2015.07, respectively, which implies that these keywords may become research hot spots of this field in coming years.

## Discussion

Different from the systematic reviews, bibliometric analysis is now an effective tool for summarizing the current status and predicting the future development trends in the knowledge domain of interest research (Grant and Booth, 2009; Møller and Myles, 2016). The visualization map created by using VOS viewer or CiteSpace is based on information science, computer science, scientometrics, and applied mathematics and displays the development process and structural relationships of knowledge in a certain field (Synnestvedt et al., 2005; van-Eck and Waltman, 2010; Gu et al., 2019). Therefore, through using bibliometric and visualized analysis, we examined patterns of research on ultrasound microbubble, identifying the main publications, contributing countries, authors, funds, research orientations, and topics addressed and measuring the contribution of different countries and institutions.

Our findings showed that the field of ultrasound microbubble had undergone tremendous expansion and the number of publications had an upward trend over the study period of 1998–2019. A total of 6,088 papers with 194,896 cited times were searched using the WoSCC. Among the 73 countries which participated in the publication of studies in this domain, the United States was the most productive country. Initially, there had been a large gap between the United States and other countries, but this gap gradually decreased over the years as the publication number in China increased. The total number of publications in China ranked second only to that in the United States, reflecting a growing interest of Chinese researchers in this area. Thus, it can be boldly predicted that more publications on ultrasound microbubble may become available in the coming years as a result of growing concern. The bibliometric results based on the Scopus database also certificated these conclusions.

H-index is an important indicator to characterize the scientific output and academic status of a researcher. For example, if an author has an H-index of n, it means that he/she has n publications that have received n citations or even more (Merigó and Yang, 2017). Thus, H-index is the dominant metric that has been used for quantifying an individual’s scholarly output. Apart from that, previous studies also considered it as a useful indicator for evaluating the productivity and impact of a country, institution, or journal (Engqvist and Frommen, 2008). Similar to H-index, the total number of citations from one country, institution, or journal could also represent the quality and academic impact of their publications. Our study showed that the United States was the leading country in both the total number of citations and H-index. From the perspective of H-index, England and Germany have also played an important role in this field. Additionally, among the top 10 most productive institutions, China accounted for three institutions and was the only developing country, indicating China’s great progress in this field. Although this is a considerable advancement, it should also be noted that China’s average citation rate was much lower than that of the United States and its H-index was ranked only fourth. Other than quantity increase, further improvements in quality of publications are warranted. In the meantime, the research and development of ultrasound microbubble requires a large amount of both human and financial resources. Apparently, economic foundation plays an important role in supporting scientific research. Among the top 10 funding agencies, most of the major contributing agencies were distributed in the United States.

For journals, *Ultrasound in Medicine and Biology* (IF = 2.514, Q1), *IEEE Transactions on Ultrasonics, Ferroelectrics and Frequency Control* (IF = 2.812, Q1), and the *Journal of Controlled Release* (IF = 7.727, Q1) published the most articles on ultrasound microbubble. These journals with high impact factors and JCR categories attract papers of high quality, and the publication of excellent papers can also in turn raise the academic influence of these journals. In the cocited network map, *Ultrasound in Medicine and Biology* had the highest centrality, followed by the *Journal of Controlled Release, Radiology, and Circulation*. Consequently, it can be speculated that future development in this field is still more likely to be published in the listed journals. In addition, a dual-map overlay, which can reveal the trends of the scientific portfolio in the overall visualization, was used as a knowledge carrier to analyze the information flow to and between journals. The results show that the published studies mainly targeted journals in the fields i) physics, materials, and chemistry; ii) molecular, biology, and immunology; and iii) medicine, medical, and clinical. These journals mostly cited journals from i) chemistry, materials, and physics; ii) molecular, biology, and genetics; and iii) health, nursing, and medicine.

Coauthorship analysis refers to the evaluation of the relationship among items through the number of coauthored documents. It was utilized to evaluate the cooperation between different authors, institutions, and countries in this study. The TLS is an indicator that can be used to quantitatively assess the closeness of cooperation. Results with a higher TLS indicate that the authors, institutions, and countries tend to work collaboratively. That is, the greater the value was, the more frequent the cooperation was. Current results suggested that the United States, as the center of research, had close collaborations with China and Canada. For the network map of institution coauthorship analysis, the University of Toronto had close collaborations with the Sunnybrook Research Institute, Sunnybrook Health Sciences Centre, and Hospital for Sick Children, and the Chinese Academy of Sciences had massive collaborations with Nanjing University, Tongji University, and Chongqing Medical University. However, the low-density map implied that the research groups were relatively dispersed in various institutions, and interinstitutional collaboration still needs to be strengthened. The centrality indexes in most institutions were less than 0.15, suggesting a lack of cooperation between institutions, and the influence of most institutions remains at a low level. For author coauthorship analysis, De Jong N from the Netherlands was the author with the most publications, followed by Dayton PA and Klibanov A L from the United States and Wang ZG from China. They and their institutions exert important influence in the research area of emerging development in ultrasound microbubble. From the view of centrality, Dayton P A, De Jong N, and Wang Z G were located at a central position of the cooperating clusters.

Cocitation analysis is a research method for measuring the degree of the relationship between documents. In the author cocitation analysis, the relevance between authors depends on the number of times that their papers are cited by the same article. This method is frequently used to evaluate the authors’ academic influence. Our results suggest that Lindner JR owned the highest centrality and was also one of the highly cited authors, which indicated that the papers written by him have high scientific impact. Lindner JR mainly engaged in research on cardiovascular system cardiology and radiology nuclear medicine medical imaging (Ellegala et al., 2003; Lindner, 2004). In the cluster map, authors’ research categories could be divided into nine clusters, such as “vascular gene transfer” (#0) (Ferrara et al., 2007; Hernot and Klibanov, 2008), “hepatocellular carcinoma” (#1) (Mulier et al., 2002), “ultrasound contrast agent microbubble” (#2) (Lindner, 2004), etc. These research directions have received the most attention from scholars in this field. As can be seen, the focus of scholars was mainly concentrated on diagnostic and therapeutic applications of ultrasound contrast microbubbles. There are also many scholars who have devoted themselves to the study of the acoustic characteristics to improve the biological properties of microbubbles.

The top 10 highly cited studies were published between 1998 and 2013, and seven studies were published prior to 2010. The most highly cited paper was written by Wei et al. (1998). The reference cocitation analysis from the two databases also confirmed the central position in the network map. The main contribution of this study was proposing that microbubbles had the potential for measuring tissue perfusion in any organ accessible to ultrasound. In addition, it is apparent that highly cited articles did not occur in recent years, which could mainly be related to the time factors. In the reference cocitation network, articles with similar topics are usually cited together and more inclined to be concentrated on one. Figure 8B shows the timeline view of the reference cocitation clusters, which can reflect the dynamic changes and development trends of the corresponding clusters in different periods. The largest cluster was “myocardial perfusion” (#0) (Wei et al., 1998; Leong-Poi et al., 2001), followed by “ultrasound-assisted drug delivery” (#1) (Dixon et al., 2015; Sennoga et al., 2017) and “focal liver lesion” (#2) (Kondo et al., 2017). The development of cluster 0 (myocardial perfusion) and cluster 11 (splenic parenchyma) occurred earliest, while cluster 1 (ultrasound-assisted drug delivery) and cluster 5 (blood–brain barrier permeability) are current research hot spots. This might imply that the research focus in this field seems to have shifted from diagnostic studies to therapeutic studies. These findings are in line with the results based on the two databases analyzed by the VOS viewer as follows.

In bibliometrics, analysis of frequently appearing keywords can also reveal the hot spot categories and the development of a research topic. According to the keyword cooccurrence analysis performed by using the VOS viewer, all the identified keywords from the WoSCC could be divided into four clusters: “ultrasound diagnosis study,” “microbubbles’ characteristics study,” “gene therapy study,” and “drug delivery study.” These four clusters represent the main research direction in the field of ultrasound microbubble. From the overlay visualization map in Figure 6B, it can be observed that the research focus has gradually shifted from “ultrasound diagnosis study” and “microbubbles’ characteristics study” to “gene therapy study” and “drug delivery study.” Similar results were also obtained by analyzing the Scopus database. It is noteworthy that the keywords related to gene therapy and drug delivery were combined into one cluster in this result, and both of them were about therapeutic applications of ultrasound microbubble. This change process of the field was in accordance with the development law of translational medicine. Advances in basic research on sonographic techniques and microbubbles are now paving the way for clinical application. Therefore, the scientific community seems to be taking a particular interest in the therapeutic potential of ultrasound microbubble research at present.

Furthermore, our data showed that keywords with the latest AAY, such as “doxorubicin,” “nanoparticles,” and “breast cancer,” may become the research hot spots in coming years.

(i) Doxorubicin. Doxorubicin, a compound of the anthracycline class, is one of the most powerful and widely used chemotherapeutic drugs in the treatment of various types of solid tumors and hematological malignancies (Escoffre et al., 2011). However, its severe side effects, including brain, heart, liver, and kidney toxicities, limit its clinical application (Carvalho et al., 2009). To overcome this problem, the development of an efficient, targeted drug delivery system for cancer cells is necessary to reduce the dose required to achieve the same therapeutic effect and potentially reduce side effects. In recent years, multiple studies have identified that the application of ultrasound combined with different types of microbubbles could enhance the intracellular delivery of drugs on cells, tissues, and even the biological barriers (Escoffre et al., 2011; Escoffre et al., 2013; Yu et al., 2016; Luo et al., 2017; Alli et al., 2018). Predictably, substantial effort and resources will be devoted to the development and testing of doxorubicin and microbubble complexes assisted by ultrasound in the future.
(ii) Nanoparticles. Although microbubble-based ultrasound imaging has its own unique advantages compared to other imaging techniques, traditional microbubbles also have certain drawbacks such as large particle size, low stability, difficult structural control, etc., which limit their application in many fields, especially extravascular (Zhou et al., 2020). Fortunately, the clipping progress of nanomedicine and biomaterial science further broadens the application values of ultrasound (De-Cock et al., 2016; Blum et al., 2017). The outstanding properties including unique structures, compositions, and corresponding multifunctionalities of nanomaterials make them ideal candidates for improving ultrasound molecular imaging and targeted therapy. For instance, they can be taken as carriers to translocate DNA/RNA (Zhao et al., 2018), drug molecules (Arosio et al., 2012), and other useful materials into cell interiors (Kato et al., 2015). Several studies have demonstrated that the introduction of designed nanoparticles into oncologic therapies could substantially enhance the therapeutic efficiency (Blum et al., 2017; Qian et al., 2017). Notably, the research in this area, especially inorganic nanoparticles, is still at a preliminary stage, but the rapid development makes this research hot spot an even more promising area for clinical transformation.
(iii) Breast cancer. Breast cancer is the leading cause of cancer death in women. In the current management of breast cancer, chemotherapy is an important treatment strategy used to eliminate cancer cells. As already mentioned, adverse effects and low drug availability of chemotherapeutic agents limit their clinical benefits. Ultrasound combined with microbubbles provides new insight into the targeted delivery of anticancer drugs (Marshalek et al., 2016; Snipstad et al., 2017; Song et al., 2019). In addition, molecularly targeted contrast-enhanced ultrasound with microbubbles is also an emerging molecular imaging tool with a large potential to improve diagnostic accuracy of conventional ultrasound in breast cancer detection (Bachawal et al., 2015; Hoyt et al., 2015).

### Strengths and Limitations

There are several notable strengths to our study. First, this study, for the first time, systematically analyzed the global research trends of ultrasound microbubbles over the last 22 years by using the scientometric method, which can provide scientific researchers with panoramic knowledge of this field and some references on research hot spots and future directions. Second, we employed two widely used scientometric software tools to conduct the present study in paralle, and could get more comprehensive and reliable analysis results. Third, unlike previous similar bibliometric studies, whose results derive from only one database, two databases (WoSCC and Scopus) were employed in this study, which could provide a comprehensive analysis of ultrasound microbubble research (Merigó et al., 2019; Wang et al., 2019; Shi et al., 2020; Chen et al., 2021).

In spite of the advantages mentioned above, several limitations should be noted. First, given the limitations of bibliometric software, it is hard to merge the two databases for analysis, and the WoSCC was selected as the main searching database, while the Scopus database provided additional data. Other large medical databases such as PubMed and Embase were also not included. However, it should be noted that the WoSCC is the most commonly used and recommended database for bibliometric analysis (Wang et al., 2019; Chen et al., 2020; Shi et al., 2020). Therefore, the bibliometric results of this study were mainly based on the WoSCC database. Second, the bibliometric analysis results might be different from the actual research situation since the WoSCC database updates the research continuously. Some recently published and potentially influential papers may not have appeared in our study due to low citation frequency. Third, only papers written in English were included, and some key research studies published may have been neglected.

## Conclusion

The current study has summarized the present research status and emerging global trends in ultrasound microbubble research. There has been an increasing amount of research and the United States is staying ahead in both the sum of publications and total citation frequency in this field. Thus, it is not difficult to predict that this area of research is likely to continue to rapidly expand and more studies will be published in the coming years. However, collaboration between research teams still needs to be strengthened. In particular, with the focus gradually shifting from “ultrasound diagnosis study” to “gene therapy study” and “drug delivery study,” studies about “doxorubicin,” “nanoparticles,” and “breast cancer” will be the next potential research hot spots. In the near future, promising research directions might attract the attention of related scientists and funding organizations and alsomight open up new ultrasound microbubble–based diagnosis and therapeutic concepts.

## Data Availability

The original contributions presented in the study are included in the article/Supplementary Material; further inquiries can be directed to the corresponding authors.
