# Supplementary material for: Bibliometric Analysis of Global Research Trends on Ultrasound Microbubble: A Quickly Developing Field
Source: Front Pharmacol. 2021 Apr 22;12:646626. doi: 10.3389/fphar.2021.646626 (PMC8101552; doi:10.3389/fphar.2021.646626)
Supplement: Supplementary file 1 [file datasheet1.docx]

Supplementary Material

# Supplementary Figures and Tables

## Supplementary Figure


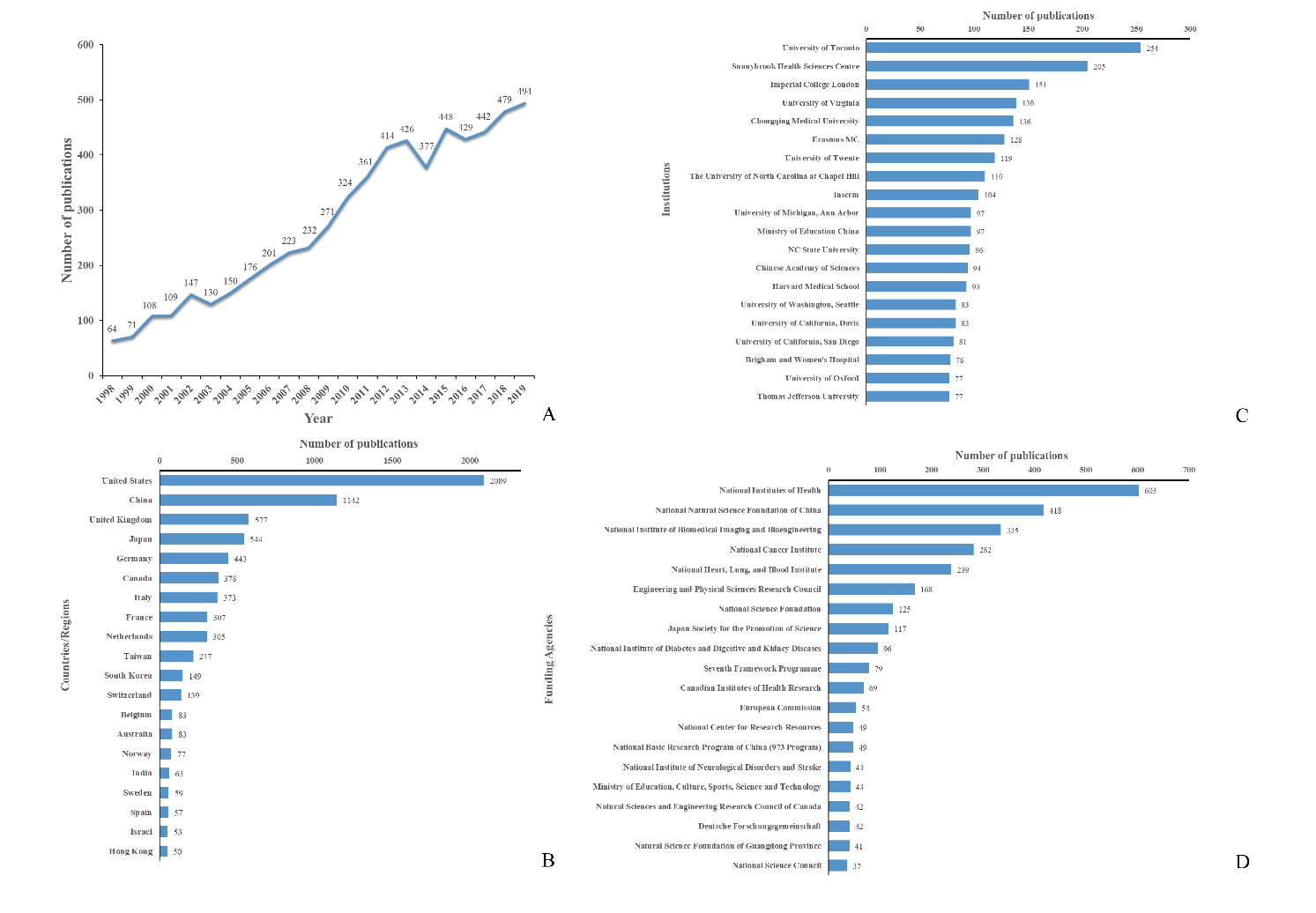


**Supplementary Figure 1.** (A) The global trend of annual publications related to ultrasound microbubble research from 1998 to 2019. A total of 6150 publications including 5367 articles and 783 reviews met the inclusion criteria from 1988 to 2019. The trend of global ultrasound microbubble research publications has been steadily increasing in the past 22 years. (B) The top 20 countries/regions contributed to the total publications. The USA was the foremost productive country, with 2089 papers published (34.0%), followed by China (1142, 18.6%). (C) The top 20 institutions contributed to the total publications. University of Toronto was the largest contributor in terms of numbers of publications with 254 papers. (D) The top 20 research funds and the number of publications in each fund. National Institutes of Health (NIH) and National Natural Science Foundation of China (NSFC) were the most contributing funding agencies. All the results of Figure A-D were based on the data obtained from Scopus database.


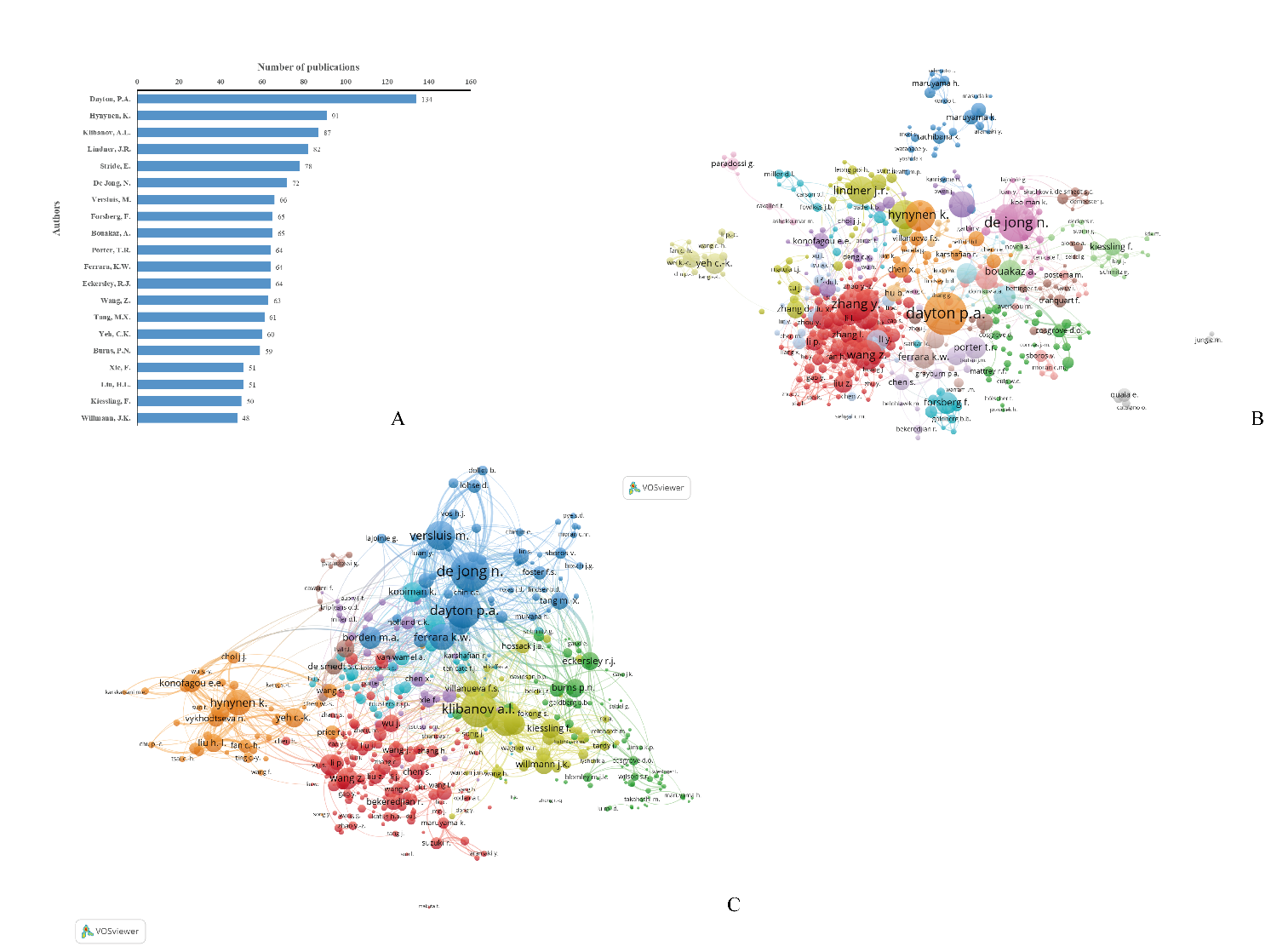


**Supplementary Figure 2.** (A) The top 20 most-productive authors based on the number of publications. Dayton P A from USA was the author with the most publications of 134. (B) Mapping of the co-authorship analysis among 503 identified authors on ultrasound microbubble research based on VOS viewer. Authors with a minimum of 10 publications were included. There were 503 nodes and 4936 links in the network map. The top 3 authors with the largest TLS were Wang Z G (TLS = 385), DE JONG N (TLS = 377), and Zhang Y (TLS = 313). (C) Co-citation map of authors on ultrasound microbubble research by VOS viewer. There were 503 nodes and 59166 links in the network map. The top 3 authors with the largest TLS were DE JONG N (TLS = 13235), Klibanov A L (TLS = 11478), and Dayton P A (TLS = 11343). All the results of Figure A-C were based on the data obtained from Scopus database.


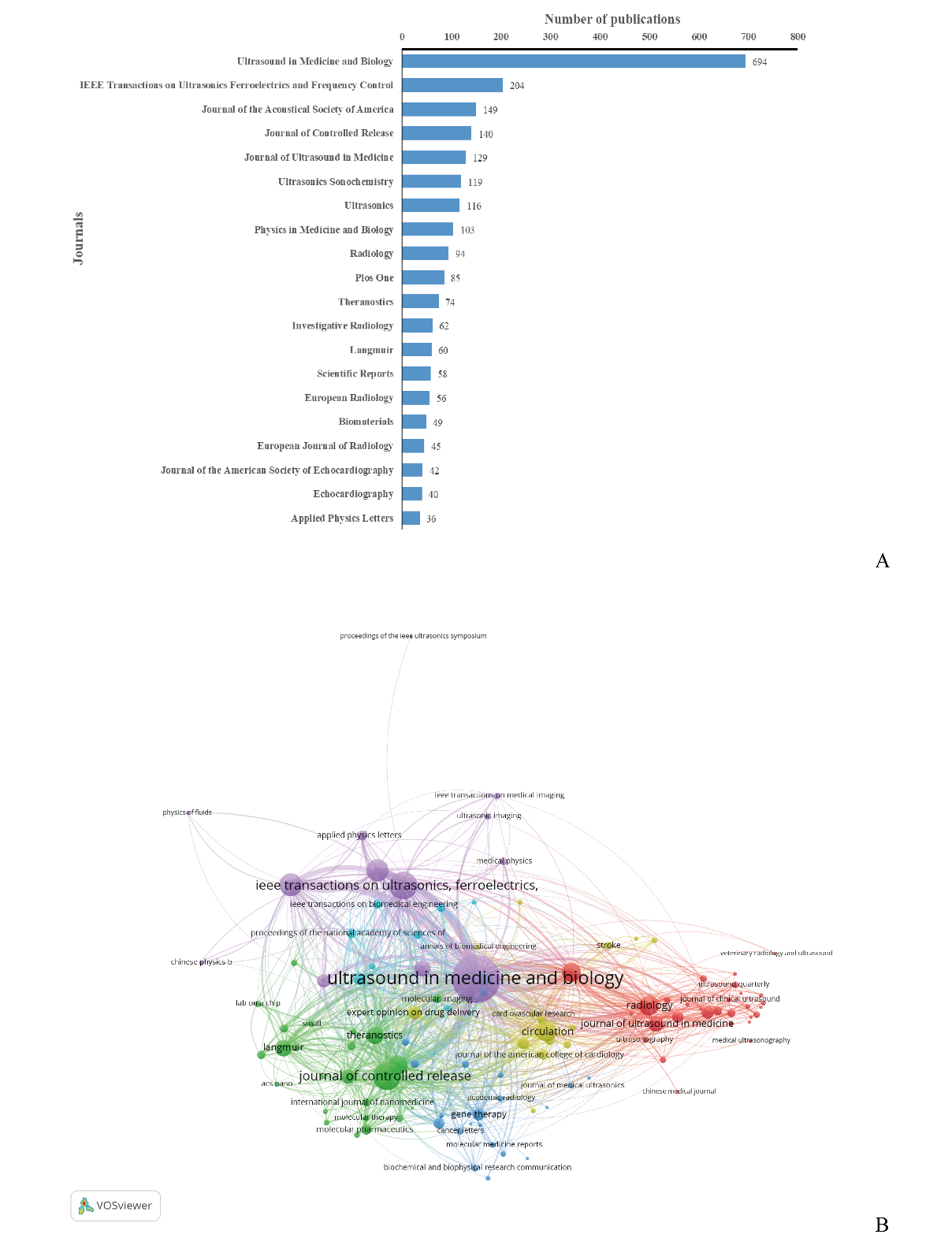


**Supplementary Figure 3.** (A) Top 20 journals in the field of ultrasound microbubble research ranked by publication number. *Ultrasound in Medicine and Biology* published the most articles/reviews (694 papers), accounting for 11.28% of the publications. (B) As for journals co-citation analysis, only institutions with a minimum of 10 publications (110 journals) were included. The top 3 with the largest TLS were *Ultrasound in Medicine and Biology* (TLS = 10845), *Journal of Controlled Release* (TLS = 3718), *IEEE Transactions on Ultrasonics*, *Ferroelectrics and Frequency Control* (TLS = 3708). All the results of Figure A-B were based on the data obtained from Scopus database.


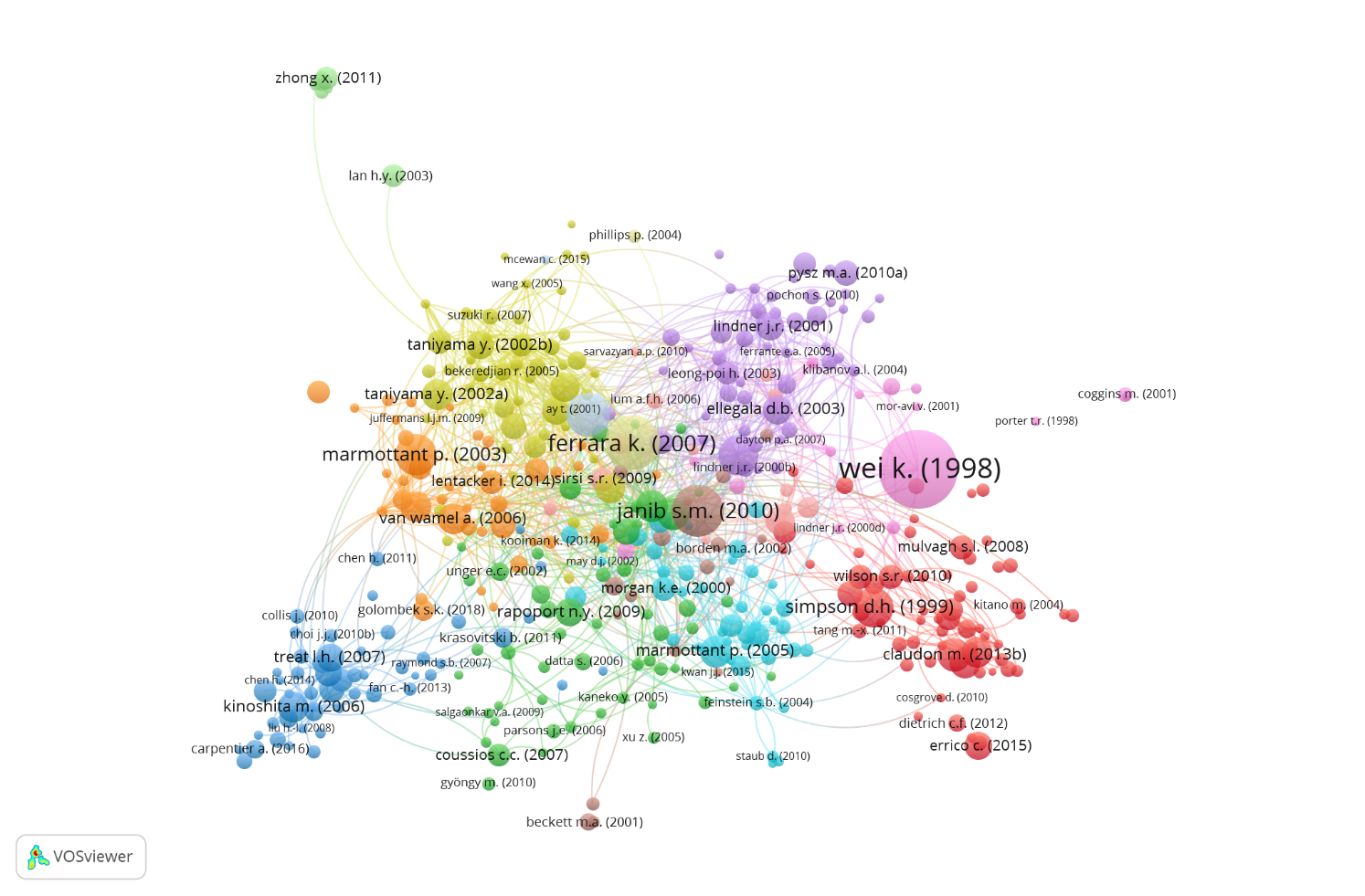


**Supplementary Figure 4.** Co-citation map of references on ultrasound microbubble research by CiteSpace. Wei K (1998), Ferrara K (2007), and Janib S M (2010) were the references with the most citations. All the results were based on the data obtained from Scopus database.


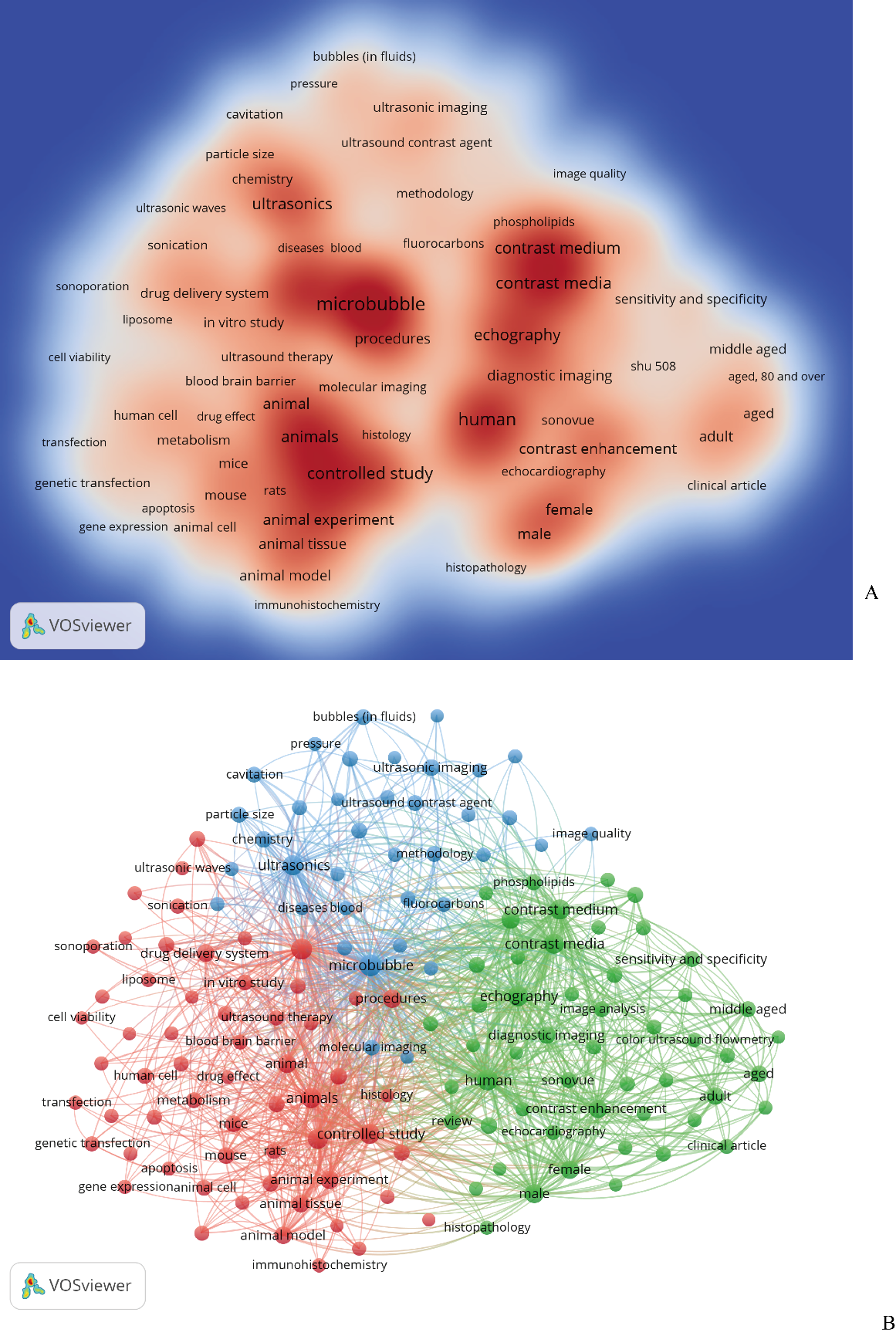


**Supplementary Figure 5.** (A) A density map of high frequency keywords was created using VOS viewer. The results revealed that there were 29006 keywords in the 6150 papers, and 315 keywords appeared 100 times or more. (B) Network visualization of the Keywords co-occurrence analysis. all the identified keywords could be divided into 3 clusters: “ultrasound diagnosis study” (green node), “microbubbles’ characteristics study” (blue node), and “gene and drug therapy study” (red node). All the results were based on the data obtained from Scopus database.
